# Supplementary material for: Bottleneck Effect Explained by Le Bail Refinements: Structure Transformation of Mg-CUK-1 by Confining H2O Molecules
Source: Materials (Basel). 2020 Apr 14;13(8):1840. doi: 10.3390/ma13081840 (PMC7215401; doi:10.3390/ma13081840)
Supplement: Supplementary file 1 [file materials-13-01840-s001.pdf]

materials

Supplementary Materials

Bottleneck Effect Explained by Le Bail Refinements: Structure Transformation of Mg-CUK-1 by Confining H_2O Molecules

Elí Sánchez-González [1,2,†], J. Gabriel Flores [1,3,4,†], Julio C. Flores-Reyes [5], Ivette Morales-Salazar [5], Roberto E. Blanco-Carapia [5], Mónica A. Rincón-Guevara [6], Alejandro Islas-Jácome [5], Eduardo González-Zamora [5,*], Julia Aguilar-Pliego [3,*] and Ilich A. Ibarra [1,*]

1 Laboratorio de Fisicoquímica y Reactividad de Superficies, Instituto de Investigaciones en Materiales, Universidad Nacional Autónoma de México, Circuito Exterior S/N, Ciudad Universitaria, Coyoacán, Ciudad de México C.P. 04510, Mexico; sanchez.gonzalez.eli@gmail.com (E.S.-G.); gabriel_flores_aguilar@hotmail.com (J.G.F.)
2 Institute for Integrated Cell-Material Sciences (WPI-iCeMS), Kyoto University, Yoshida, Sakyo-ku, Kyoto 606-8501, Japan
3 Departamento de Química Aplicada, Universidad Autónoma Metropolitana-Azcapotzalco, San Pablo 180, Col. Reynosa-Tamaulipas, Azcapotzalco, Ciudad de México C.P. 02200, Mexico
4 Instituto de Catálisis y Petroleoquímica, ICP-CSIC, C/ Marie Curie, 2, C.P. 28049 Madrid, España
5 Departamento de Química, Universidad Autónoma Metropolitana-Iztapalapa, San Rafael Atlixco 186, Col. Vicentina, Iztapalapa, Ciudad de México C.P. 09340, Mexico; flores.reyes.jc@gmail.com (J.C.F.-R.); ivette649_tatu@hotmail.com (I.M.-S.); edreyblanco@gmail.com (R.E.B.-C.); aij@xanum.uam.mx (A.I.J.)
6 Departamento de Biotecnología, Universidad Autónoma Metropolitana-Iztapalapa, San Rafael Atlixco 186, Col. Vicentina, Iztapalapa, Ciudad de México C.P. 09340, Mexico; monicarinconguevara@gmail.com
* Correspondence: egz@xanum.uam.mx (E.G.-Z.); apj@correo.azc.uam.mx (J.A.-P.); argel@unam.mx (I.A.I)
† Both authors contributed equally to this work.

Received: 12 January 2020; Accepted: 8 April 2020; Published: 14 April 2020

Experimental Section

Chemicals. 2,4-Pyridinedicarboxlic acid, magnesium nitrate hydrate ($Mg(NO_3)_2$) and potassium hydroxide (KOH) were obtained from Sigma-Aldrich and used as received.

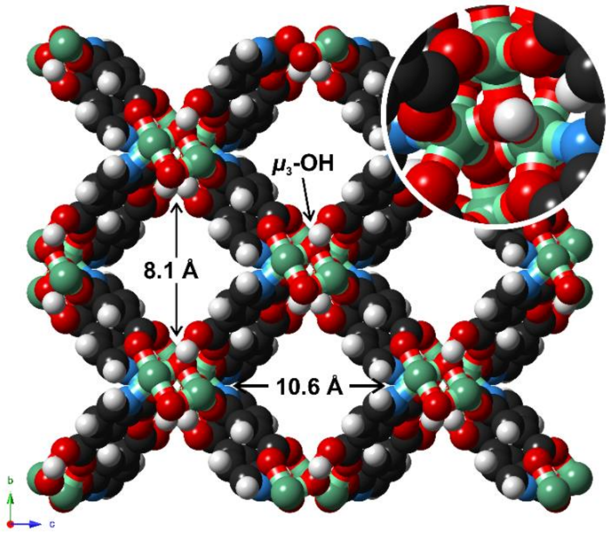

Figure S1. Crystal structure of Mg-CUK-1 viewed through the *a*-axis, depicting the one-dimensional channels with the hydroxyl group in the *b* direction. Inset, trinuclear Mg(II) building block with the hydroxyl group pointing towards the center of the channel.

Characterization of Mg-CUK-1

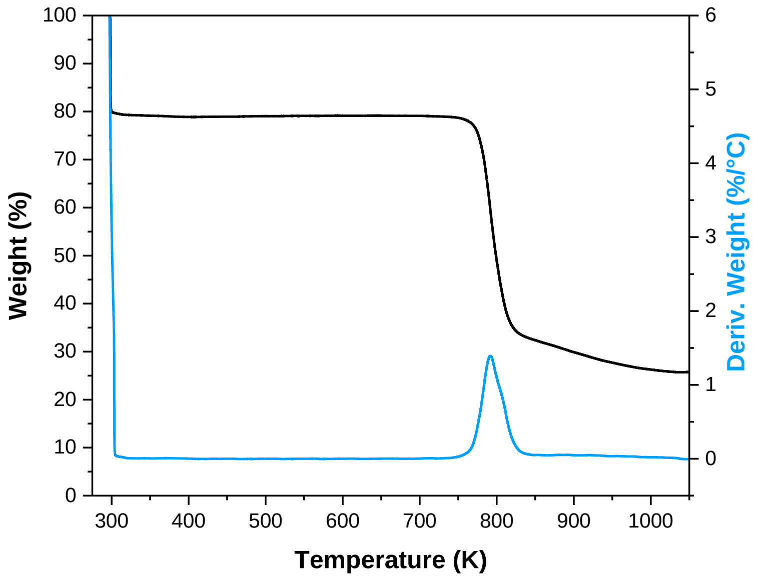

Figure S2. Thermogravimetric analysis profile of Mg-CUK-1 as synthesized, under N_2 atmosphere.

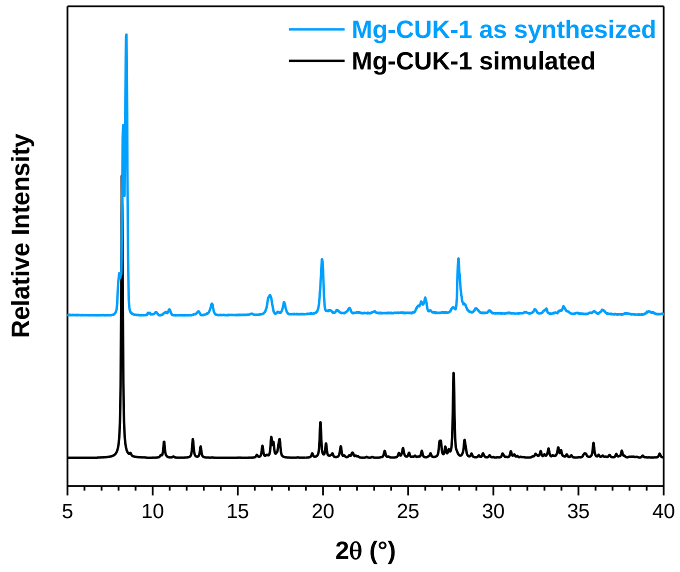

Figure S3. PXRD pattern of Mg-CUK-1 as synthesized (blue trace) and simulated (black trace). The reported hydrated Mg-CUK-1 structure was used for the simulated pattern (CCDC structure NUDLIJ).

PXRD Profile Refinement of Mg-CUK-1

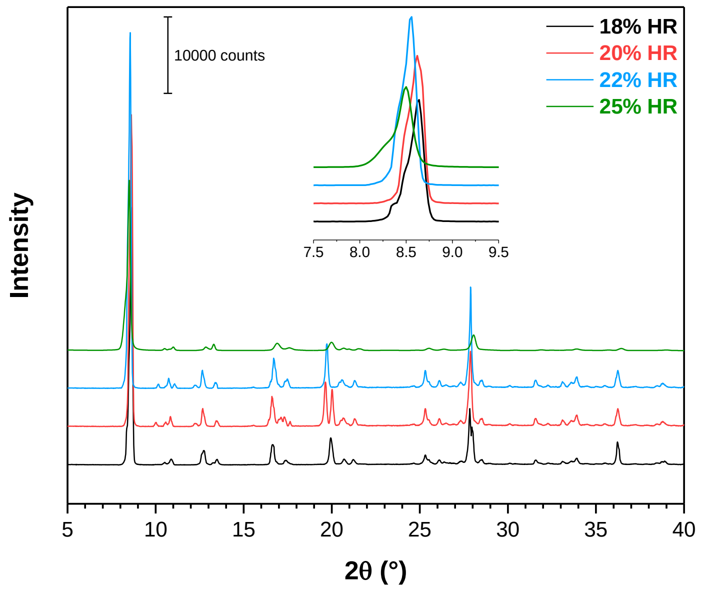

Figure S4. Comparison of Mg-CUK-1 PXRD patterns loaded at different relative humidity values: 18%, 20%, 22% and 25% RH. Inset shows 011 plane reflection.

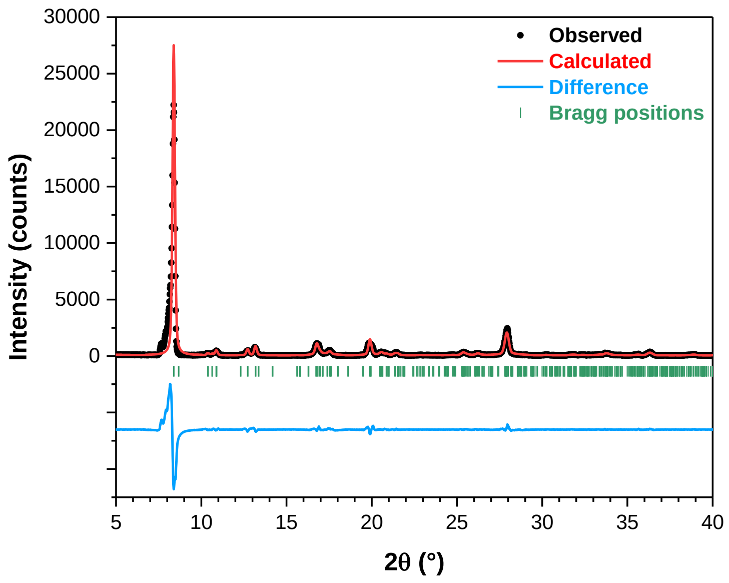

Figure S5. Profile refinement of the Mg-CUK-1 PXRD pattern loaded at 25% RH, approximately 8 water molecules per unit cell.

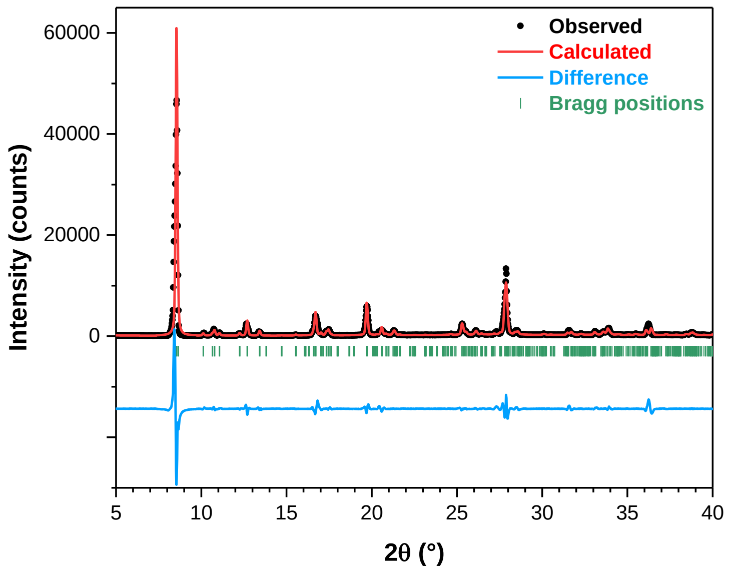

Figure S6. Profile refinement of the Mg-CUK-1 PXRD pattern loaded at 22% RH, approximately 4 water molecules per unit cell.

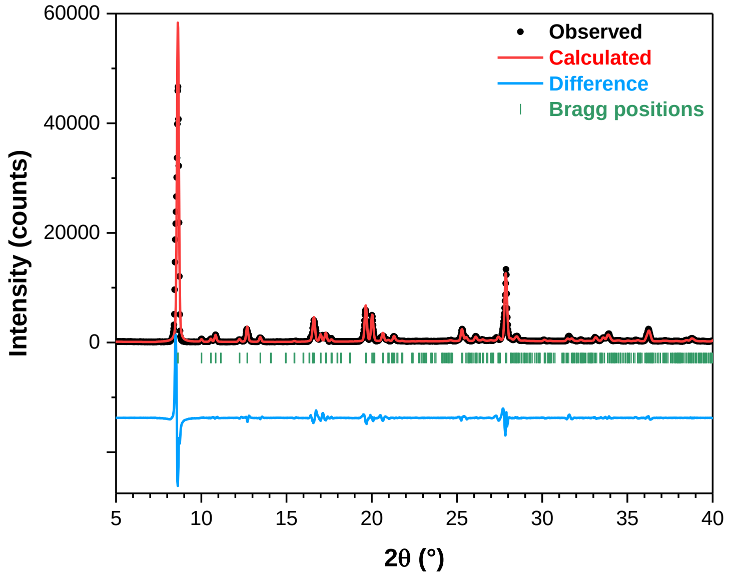

Figure S7. Profile refinement of the Mg-CUK-1 PXRD pattern loaded at 20% RH, approximately 2 water molecules per unit cell.

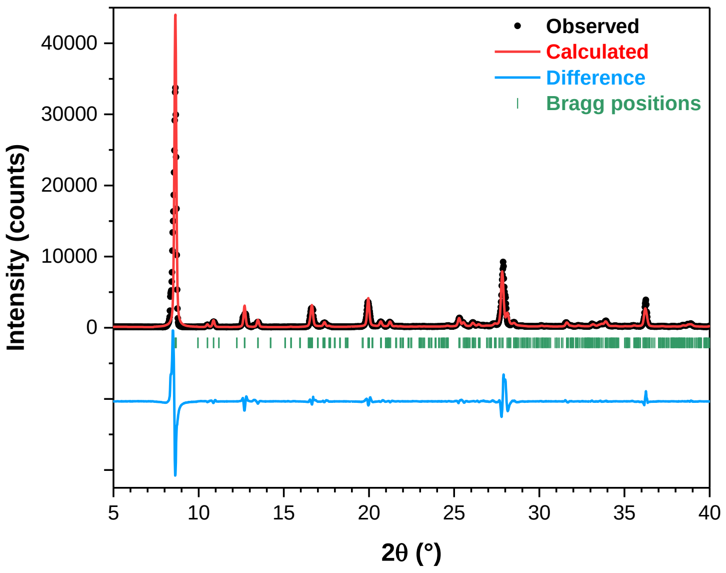

Figure S8. Profile refinement of the Mg-CUK-1 PXRD pattern loaded at 18% RH, approximately 1 water molecules per unit cell.

The Le Bail profile refinements of the Mg-CUK-1 samples at different water loadings (18%, 20%, 22% and 25% RH) were performed to obtain the cell parameters of each sample (Figures S4–S7). Refinements were performed based on the previously reported Mg-CUK-1-hydrated data (CCDC structure NUDLIJ) [1]. Although the goodness-of-fit parameters (χ^2 and *R* factors) are not what is expected for a "good fit" [2] (Table S1), these high values could be explained by the presence of more than one phase in the pattern. The PXRD measurements were performed at room temperature with no control over the relative humidity. Since Mg-CUK-1 is hydrophilic above 30% RH, it is plausible to have a small amount of a "more" hydrated phase in the surface of the sample. This should be reflected in an extra reflection at lower two theta angles, accounting for the marked difference in the first reflection around 8.5° (Figure S4–S7), and thus the high values for the goodness-of-fit parameters.

Table S1. Refinement parameters of the Mg-CUK-1 structure at different water loadings.

**Formula**	**$Mg_3(OH)_2(C_7H_3O_4N)_2{\cdot}XH_2O$**				
**X =**	**2**	**1**	**0.5**	**0.25**	**0 ***
**H_2O per UC**	8	4	2	1	-
**Crystal system**	monoclinic	monoclinic	monoclinic	monoclinic	monoclinic
**Space group**	$P2_1/n$	$P2_1/n$	$P2_1/n$	$P2_1/n$	$C2/c$
**a (Å)**	10.9329	10.9830	11.0034	11.0135	18.293
**b (Å)**	13.4350	12.8277	12.5809	12.4574	12.3340
**c (Å)**	17.5753	17.9688	18.1304	18.2116	11.0237
**β (°)**	104.351	103.305	102.893	102.690	102.488
**V (Å³)**	2500.98	2463.61	2446.56	2437.59	2428.39
**R_{wp} (%)**	57.0	50.1	44.2	52.6	-
***R* (%)**	45.1	36.1	32.1	38.7	-
**χ^2**	51.4	89.1	72.7	74.7	-

* These parameters correspond to the reported de-hydrated structure (CCDC structure NUDLOP01).

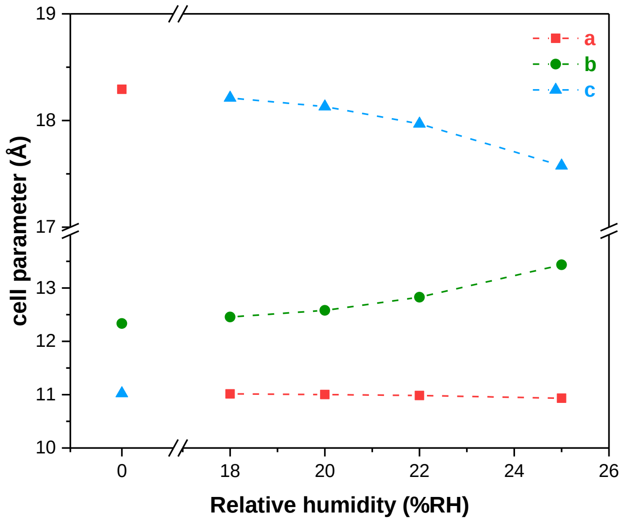

Figure S9. Comparison of the obtained cell parameter for the Mg-CUK-1 loaded at different relative humidity values: 18%, 20%, 22% and 25% RH. The cell parameters of the de-hydrated sample are included at 0% RH, these parameters were taken from a different structure (structure NUDLOP01) than the one the refinements were based on (structure NUDLIJ).

Using the obtained cell parameters, plausible crystal structures were constructed, by taking the reported Mg-CUK-1-hydrated structure (CCDC structure NUDLIJ) and changing the cell parameters [1]. The excess of water molecules was removed, leaving only the ones placed over the hydroxo groups within the channel. The proposed crystal structures were obtained by removing the water molecules to leave almost evenly-spaced water molecules in the unit cell (Figure S8), matching the water loadings of the experimental data (Table S1).

The distances between the water molecules were measured from the O_{H2O}-O_{H2O} and by subtracting the oxygen van der Waals radius (1.52 Å) (Figure S8). Conversely to the data of Table S1, the length of the minimum distance in the *b*- and *c*-axis was measured from the void obtained with a 0.002 au isosurface with CrystalExplorer. This calculus considers the hydrogen atoms to calculate the void, thus these values are expected to be smaller.

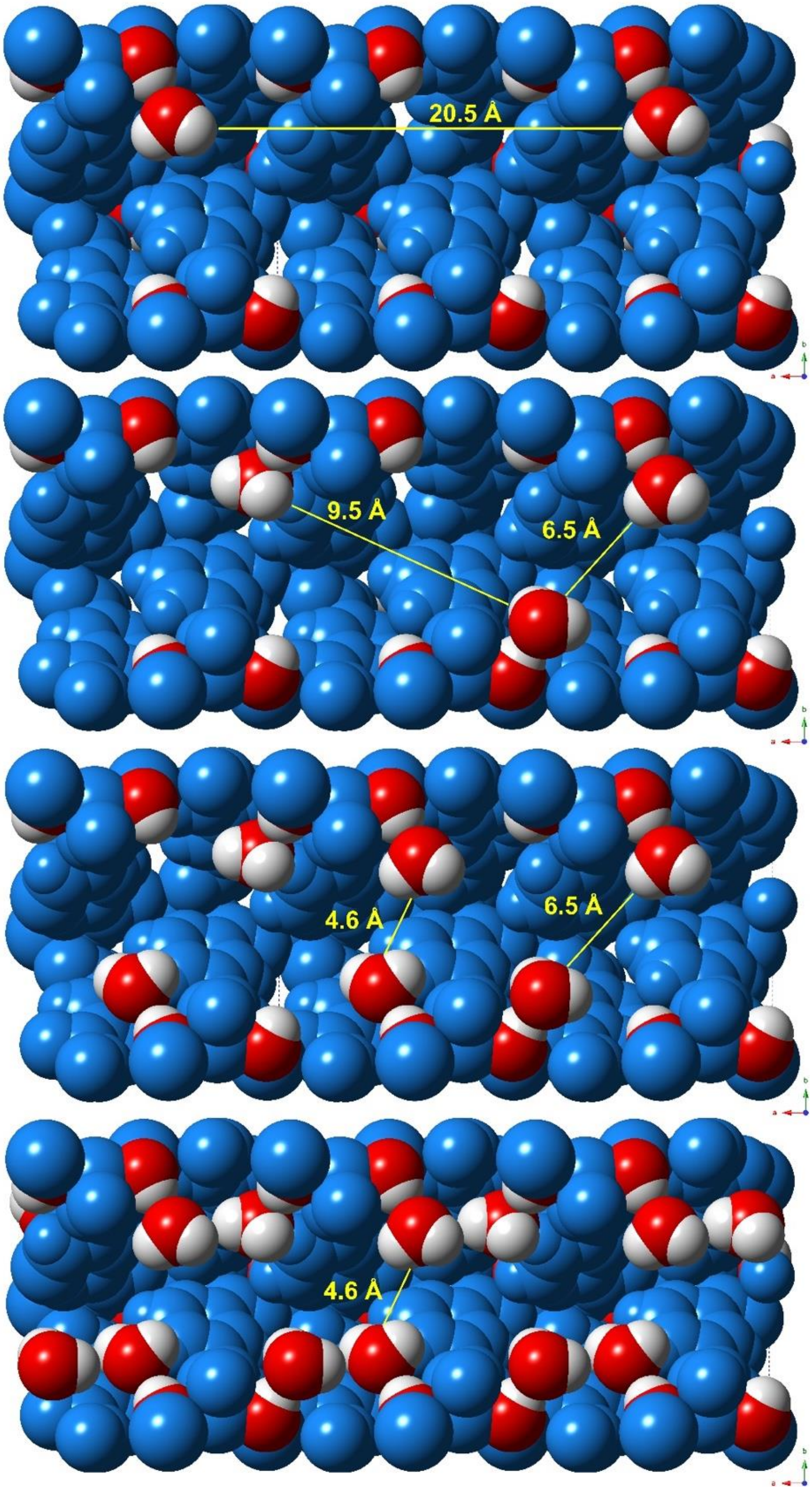

Figure S10. Side view of the channel through the *c*-axis, marking the distance between adjacent water molecules at different H_2O loadings (from top to bottom 1, 2, 4 and 8 H_2O per unit cell).

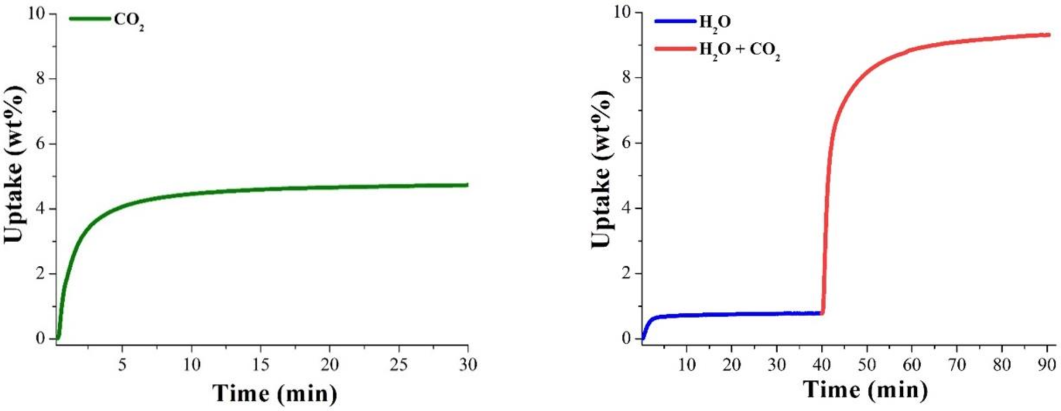

(**a**) (**b**)

Figure S11. (**a**) Kinetic CO_2 uptake experiment performed at 303 K with a CO_2 flow of 60 mL min^{-1}; (**b**) kinetic CO_2 uptake experiments carried out at 18% RH at 303 K; H_2O (blue line) and H_2O+CO_2 (red line) [3].

References

1. Saccoccia, B.; Bohnsack, A.M.; Waggoner, N.W.; Cho, K.H.; Lee, J.S.; Hong, D.-Y.; Lynch, V.M.; Chang, J.-S.; Humphrey, S.M. Separation of *p*-Divinylbenzene by Selective Room-Temperature Adsorption Inside Mg-CUK-1 Prepared by Aqueous Microwave Synthesis. *Angew. Chem.* **2015**, *127*, 5484–5488.
2. Toby, B.H. R Factors in Rietveld Analysis: How Good Is Good Enough? *Powder Diffr.* **2006**, *21*, 67–70.
3. Sagastuy-Breña, M.; Mileo, P.G.M.; Sánchez-González, E.; Reynolds, J.E.; Jurado-Vázquez, T.; Balmaseda, J.; González-Zamora, E.; Devautour-Vinot, S.; Humphrey, S.M.; Maurin, G.; et al. Humidity-induced CO_2 capture enhancement in Mg-CUK-1. *Dalt. Trans*. **2018**, 47, 15827–15834.

© 2020 by the authors. Licensee MDPI, Basel, Switzerland. This article is an open access article distributed under the terms and conditions of the Creative Commons Attribution (CC BY) license (http://creativecommons.org/licenses/by/4.0/).
